# SOX2 as a New Regulator of HPV16 Transcription

**DOI:** 10.3390/v9070175

**Published:** 2017-07-05

**Authors:** Imelda Martínez-Ramírez, Víctor del-Castillo-Falconi, Irma B. Mitre-Aguilar, Alfredo Amador-Molina, Adela Carrillo-García, Elizabeth Langley, Alejandro Zentella-Dehesa, Ernesto Soto-Reyes, Alejandro García-Carrancá, Luis A. Herrera, Marcela Lizano

**Affiliations:** 1 Unidad de Investigación Biomédica en Cáncer, Instituto Nacional de Cancerología (INCan)/Instituto de Investigaciones Biomédicas, Universidad Nacional Autónoma de México (UNAM), Ciudad de México 14080, Mexico; immara02@yahoo.com.mx (I.M.-R.); vdcfalconi@gmail.com (V.d.-C.-F.); aamadorm@incan.edu.mx (A.A.-M.); adcarrillo2004@yahoo.com.mx (A.C.-G.); langleyemx@gmail.com (E.L.); epigenetics.cancer@gmail.com (E.S.-R.); carranca@biomedicas.unam.mx (A.G.-C.); herreram@biomedicas.unam.mx (L.A.H.); 2 Unidad de Bioquímica, Instituto Nacional de Ciencias Médicas y Nutrición Salvador Zubirán (INCMNSZ)/Unidad Periférica del Instituto de Investigaciones Biomédicas, Universidad Nacional Autónoma de México (UNAM), Ciudad de México 14080, Mexico; irma.mitre@gmail.com (I.B.M.-A.); azentell@biomedicas.unam.mx (A.Z.-D.); 3 Departamento de Medicina Genómica y Toxicología Ambiental, Instituto de Investigaciones Biomédicas, Universidad Nacional Autónoma de México (UNAM), Ciudad de México 04510, Mexico

**Keywords:** human papillomavirus, HPV, LCR, SOX2, transcriptional regulation

## Abstract

Persistent infections with high-risk human papillomavirus (HPV) constitute the main risk factor for cervical cancer development. HPV16 is the most frequent type associated to squamous cell carcinomas (SCC), followed by HPV18. The long control region (LCR) in the HPV genome contains the replication origin and sequences recognized by cellular transcription factors (TFs) controlling viral transcription. Altered expression of *E6* and *E7* viral oncogenes, modulated by the LCR, causes modifications in cellular pathways such as proliferation, leading to malignant transformation. The aim of this study was to identify specific TFs that could contribute to the modulation of high-risk HPV transcriptional activity, related to the cellular histological origin. We identified sex determining region Y (SRY)-box 2 (SOX2) response elements present in HPV16-LCR. SOX2 binding to the LCR was demonstrated by in vivo and in vitro assays. The overexpression of this TF repressed HPV16-LCR transcriptional activity, as shown through reporter plasmid assays and by the down-regulation of endogenous HPV oncogenes. Site-directed mutagenesis revealed that three putative SOX2 binding sites are involved in the repression of the LCR activity. We propose that SOX2 acts as a transcriptional repressor of HPV16-LCR, decreasing the expression of *E6* and *E7* oncogenes in a SCC context.

## 1. Introduction

Cervical cancer (CC) is the third most common cause of cancer death in women from Latin America [1]. This type of cancer commonly presents as squamous cell carcinoma (SCC), corresponding to 80% of all cervical cancers, followed by adenocarcinoma (AD), with a glandular origin, which accounts for approximately 15% of the cases [2]. Persistent infection with high-risk Human papillomavirus (HPV) is the main risk factor for development of cervical cancer [3,4]. An etiological role for HPV in other types of cancer has also been reported, such as non-cervical anogenital cancer [5,6], head and neck squamous carcinoma [7] and oropharyngeal cancers [8]. More than 170 viral types have been described, with 10% of genomic differences among them [9]. About 40 HPV types infect the anogenital mucosa, and are characterized as high-risk, associated with malignant transformation, or low-risk, which cause benign warts and premalignant lesions [10,11]. HPV16 and 18 are the most frequent viral types associated with SCC (nearly 60% and 16%, respectively) [12]. However, in cervical adenocarcinoma, the proportion of HPV18 positive cases is comparable to that of HPV16, or even increases to double [12,13,14,15].

The HPV double-stranded DNA genome contains a long control region (LCR) or upstream regulatory region (URR) located between the last codon of the *L1* gene and the beginning of *E6*. This sequence contains response elements that are recognized by regulatory proteins such as transcriptional activators, repressors, terminators and initiators of replication [16,17,18,19], as well as an early promoter in the 3′ terminus (P97 for HPV16 and P105 for HPV18) [20] which mainly regulates transcription of the early (E) viral region, encoding six open reading frames. Viral proteins E1 and E2 are necessary for viral genome replication; E4 has a role in virion release; E5 activates the epidermal growth factor receptor (EGFR) [21]; and E6 and E7 are the most important viral oncoproteins [22]. Altered expression of E6 and E7 oncoproteins may cause modifications in critical cellular pathways involved in cell proliferation and apoptosis, leading to malignant transformation [23].

The LCR contains an “epithelial-specific” enhancer, encompassing several regulatory elements that hyper-activate viral transcription [24]. Transcription factors (TFs) that activate the LCR have been thoroughly characterized, including AP-1, NF1, Oct1, SP1 and YY1, which can activate and/or repress viral transcription [25]. Likewise, some TFs such as EPOC-1/Skn-1a, C/EBP-α, -β, c-Myb, NFATx, Pax5, and WT1, have been reported to be involved in differentiation dependent expression of early and late viral genes, [26]. Moreover, LCRs of different HPV types contain distinctive potential TF binding sites, which could be associated to viral expression patterns associated to each viral type, in response to host-cell differentiation [27]. For example, it has been shown that GATA3 and TFAP2B specifically affect HPV18 transcription, while no such binding sites are present in the HPV16-LCR [28].

The identification of additional TFs regulating the LCR could widen the knowledge of HPV type-specific biological behavior during the development of cervical cancer. The aim of this study was to identify specific TFs that could contribute to the modulation of high-risk HPV transcriptional activities, related to the cellular histological origin. Through in silico analysis of the HPV16- and HPV18-LCRs we identified a variety of response elements exclusive for each LCR. Interestingly, three putative sex determining region Y (SRY)-box 2 (SOX2) response elements were present in HPV16-LCR. SOX2 binding to these sites was demonstrated by in vivo and in vitro assays. The overexpression of this TF repressed HPV16-LCR transcriptional activity, as shown through reporter gene plasmid assays and by the down-regulation of the expression of endogenous HPV oncogenes. Site-directed mutagenesis revealed that the three putative SOX2 binding sites are involved in the repression of the LCR activity. We propose that SOX2 acts as a transcriptional repressor of HPV16-LCR, modulating *E6* and *E7* oncogene expression in a squamous cell context.

## 2. Materials and Methods

### 2.1. Cell Culture

CaSki HPV16 positive SCC cell line (American Type Culture Collection, ATCC # CRL-1550) was grown in RPMI-1640 medium (Life Technologies, Gibco, Grand Island, NY, USA), with 10% fetal bovine serum (FBS) (Life Technologies, Gibco). HeLa HPV18 positive adenocarcinoma cell line (ATCC # CCL-2) and the non-tumorigenic human keratinocyte HaCaT cell line (kindly donated by Dr. P. Gariglio, Centro de Investigación y de estudios Avanzados del Instituto Politécnico Nacional, CINVESTAV, Mexico), were grown in Dulbecco’s Modified Eagle Medium: Nutrient Mixture F-12 (DMEM-F12) medium (Life Technologies, Gibco), with 10% FBS, at 37 °C in a moist atmosphere of 95% air and 5% CO_2_. CaSki, HeLa and HaCaT cell lines were authenticated by short tandem repeat profiling in August 2016, by the Instituto Nacional de Medicina Genómica (INMEGEN), México and exhibited no evidence of cross contamination with known ATCC cell lines.2.2. Plasmid Constructs.

The HPV16-LCR was PCR-amplified from HPV16 positive cervical cancer biopsies. Primers used for HPV16-LCR amplification were as follows: Forward (Fwd) SmaI 5′-GGGCCCGGGCAGACCTAGATCAGTTTCCT-3′, and Reverse (Rev) HindIII 5′-GGG AAGCTTGCAGTTCTCTTTTGGTGCAT-3′, these amplify the region comprising nucleotides 7007–7905 to 1–102 of the HPV16 genome. PCR products were inserted into a SmaI/HindIII-treated pGL2-Basic vector (Promega corporation, Madison, Wisconsin, USA), which harbors luciferase gene as reporter, generating the pGL2-LCR16 vector. The pGL2-LCR18 was previously built from PCR-amplified LCR products obtained from HPV18 positive cervical cancer biopsies [29]; this plasmid contains nucleotides 7201–7857 to 1–124 of HPV genome. Plasmid constructs were verified by DNA sequencing. Plasmids pcDNA-SOX2 (Addgene, Cambridge, MA, USA) and pcDNA3.1 Zeo(+) (Life Technologies, Invitrogen, Carlsbad, CA, USA) were used for SOX2 overexpression and as a negative control, respectively.

The lentiviral vectors Pax2 and PMD2.G (Addgene) were used to produce recombinant lentiviruses containing SOX2 and/or green fluorescent protein (GFP) genes. The packaging cell line 293T was co-transfected with lentiviral expression plasmids SOX2 and/or GFP, Pax2, and PMD2.G, using lipofectamine plus (Invitrogen). Two days after transfection, cell supernatants containing the recombinant lentiviruses, were collected and stored at −80 °C until use.

### 2.3. Infection with Recombinant Lentiviruses

CaSki cells were seeded in 24-well plates at a cell density of 3 × 10^4^ cell/well. After 24 h, based on standard curves, cells were transduced with 50 microliters of lentiviral supernatant added to each well and incubation was continued for 72 h. Lentiviral vector GFP was used as transduction control.

### 2.4. Transfection Assays and Transcriptional Activity

For transient transfection assays, 30,000 CaSki, HeLa or HaCaT cells were seeded in 24-well plates with complete medium; after 24 h the cells were transfected using lipofectamine 2000 (Invitrogen) with 200 ng of luciferase reporter vector and 1 ng of Renilla plasmid (pRL-CMV) for normalization. As a positive control pGL3-Control, containing simian virus 40 (SV40) promoter was used. Cell extracts were prepared 48 h post-transfection and assayed for luciferase and renilla activities using the Dual Luciferase kit (Promega corporation, Madison, WI, USA). Relative luciferase units were measured in a Monolight^TM^ 3010 luminometer (Pharmingen, BD Biosciences, San José CA, USA). Experiments were performed in triplicate.

### 2.5. In Silico Analysis

Putative TFs binding sites within the LCR sequences of HPV16 and HPV18 were analyzed using the JASPAR CORE database [30] and alignment with consensus sequences. The complete LCR sequences of HPV16 and 18 were captured and compared with the existing data.

### 2.6. Electrophoretic Mobility Shift Assay

The Electrophoretic Mobility Shift Assay (EMSA) was performed in a final volume of 20 μL containing nuclear protein extract from HeLa cells (15 μg), in the incubation buffer (30 mM Tris pH 7.5, 300 mM KCl, 15 mM MgCl2, and 30% glycerol), 1M dithiothreitol (DTT), 10 μg/μL BSA, 1 μg/μL poly (dI-dC) (Amersham Biosciences, Buckinghamshire, UK) and 1 μL of double-stranded γ-ATP (^32^P) end-labelled DNA oligonucleotides. Primers used to identify SOX2 binding sites were: SOX2 consensus binding sequence (CS): 5′ TAGTCATACAATGTTCATTT 3′; putative SOX2 binding site 1 (S1): 5′-AACCATTCCATTGTTTTTTA-3′; putative SOX2 binding site 2 (S2): 5′-TAGTCATACATTGTTCATTT-3′; putative SOX2 binding site 3 (S3): 5′-AACTGCACATGGGTGTGTGCAAACCGT-3′; and non-specific oligonucleotide (NS): 5′ AACTGCACATGGGTGTGTGCAAACCGT 3′. The unlabeled double-stranded competitor was also included prior to the addition of labeled oligonucleotides, when specified. For the super-shift analysis, rabbit anti-SOX2 antibody (Santa Cruz Biotechnology, Dallas, TX, USA, sc-20088) and rabbit anti-CCCTC-binding factor (CTCF) (sc-5916X) were used. Binding reactions were incubated for 20 min at room temperature. Protein-DNA complexes were separated from free oligonucleotide on a 6% non-denaturing polyacrylamide/Tris borate–ethylenediaminetetraacetic acid (EDTA) gel, and were resolved at 130 V for 2 h. The gels were visualized in a Typhoon 9400 (GE Healthcare, Uppsala, Sweden) and analyzed using the Image Quant software (Bio-sciences AB GE Healthcare Life Sciences; Uppsala, Sweden).

### 2.7. Chromatin Immunoprecipitation Assay

The chromatin immunoprecipitation (ChIP) assay with CaSki cells was carried out using the OneDay ChIP kit (Diagenode, Denville, NJ, USA), following the manufacturer’s instructions. 1 × 10^6^ cells were cross-linked with 1% formaldehyde and quenched with 0.125 M of glycine. Cells were lysed with a buffer containing 1% sodium dodecyl sulfate (SDS) and sonicated with Thermo Fisher Scientific sonic disruptor (Thermo Fisher scientific, Waltham, MA, USA, Model 505). The fragmented chromatin (mainly 300 to 500 pb) was immunoprecipitated with: rabbit anti-SOX2 (Abcam, Cambridge, MA, USA, ab59776); rabbit anti-p65 (Santa Cruz Biotechnology, sc-109), as LCR-binding control [31]; and rabbit anti-CTCF (Millipore, Billerica, MA, USA, 07-729), as non-binding control [32]. As a background control, a chromatin fraction was incubated with normal rabbit IgG antibody (Santa Cruz Biotechnology, sc-2027).

Quantitative PCR (qPCR) was performed in triplicate in a fast optical 96-well qPCR reaction plate (Applied Biosystems, Carlsbad, CA, USA). Reactions were performed with primers amplifying a LCR region using the following primers: Fwd 5′-GTAAAACTACACATGGGTGTGTG-3′ and Rev 5′-GTCTGCTTTTATACTACACCGG-3′; with Thermo Maxima SYBR Green/ROX1 PCR Master Mix (Thermo Scientific, Applied Biosystems, Woolston, Warrington, UK, K0222), in StepOnePlus Real-Time PCR System (Applied Biosystems, 4376600). As a positive control of SOX2 interaction, a fragment of the Lefty promoter was PCR amplified with the following oligonucleotides: Fwd 5′-GTCAGGGTAGGGATCACTC-3′; and Rev 5′-TACAATGGTCTGAAGTGTTGC-3′.

### 2.8. Multi-Site-Directed Mutagenesis Assays

Plasmid pGL2-LCR16 was mutated in the three putative binding SOX2-sites through site-directed mutagenesis (QuikChange Multi-Site-Directed Mutagenesis kit, Agilent Technologies, USA), with primers shown in Table S1. Four different mutants were obtained: LCR-Site 1 mutant (M1), LCR-Site 2 mutant (M2), LCR-Site 3 mutant (M3) and the LCR triple mutant (M1,2,3) which is mutated in all SOX2 putative sites. LCR mutants were confirmed by direct sequencing. Site 1 was mutated in two bases, while sites 2 and 3 were mutated in three bases each (Figure S1).

### 2.9. Western Blots

Whole cell lysates were collected using RIPA buffer (150 mM sodium chloride, 0.1% Triton-X 100, 0.5% sodium dodecyl sulphate and 50 mM Tris pH8 in ddH_2_O) and prepared in the presence of complete mini EDTA-free protease inhibitor tablets (Roche, Mannheim, Germany). Protein concentrations were determined following the instructions of bicinchoninic acid solution and Copper(II) sulfate solution (Sigma-Aldrich, St. Louis, MO, USA). 30 μg of protein loaded for each lane were resolved in 12% sodium dodecyl sulfate polyacrylamide gel electrophoresis (SDS-PAGE) and transferred to an Immobilon-P polyvinylidene difluoride (PVDF) membrane (Amersham, UK) for western blot. The membranes were blocked with 10% skim milk in TBS-T (10 mM Tris pH 7.5, NaCl 100 mM and 0.1% Tween-20) and incubated with primary antibodies (in 5% skim milk) overnight at 4 °C. Protein levels of SOX2 and Lefty were analyzed with rabbit anti-SOX2 (Abcam, ab59776) (1:2000 dilution) and rabbit anti-Lefty antibodies (Santa Cruz Biotechnology, sc-48836) (1:1000 dilution). Mouse anti-α-actinin, anti-β-actin and anti-GAPDH (1:1000 dilution) (Santa Cruz Biotechnology, sc-17829, sc-47778, sc-32233, respectively), were used as convenient for protein load control. Secondary antibodies anti-rabbit or anti-mouse (1:10,000 dilution) (Santa Cruz Biotechnology, sc-2030; sc-2005, respectively), conjugated to HRP (horseradish peroxidase-conjugated), were used. Proteins were detected using Immobilon Western chemiluminescent HRP Substrate kit (Merck Millipore, Billerica, MA, USA, WBKLSO500).

### 2.10. RNA Isolation and cDNA Synthesis

Cells were resuspended in 800 μL of Trizol Reagent (Life Technologies, Invitrogen). RNA was isolated according to the manufacturer’s protocol. RNA pellets were dissolved in 20 μL DNAse-free H_2_O. RNA was quantified and treated with amplification grade DNase I (Invitrogen). cDNA was synthesized using the SuperScript II RNAseH-Reverse Transcriptase (Life Technologies, Invitrogen).

### 2.11. Quantitative PCR

cDNA generated from transfected cells was submitted to qPCR using SYBR green PCR master mix (Applied Biosystems, 4309155), according to the manufacturer’s protocol. The oligonucleotides used were for HPV16 E6, Fwd 5′-GGGGGATCCATGTTTCAGGACCCACAGGAGCGA-3′, and Rev 5′-GGGGAATTCTTACAGCTGGGTTTCTCTACGTGT-3′; for HPV16 E7, Fwd 5′-GGGGGATCCATGCATGGAGATACACCTACATTGC-3′, and Rev 5′-GGGAAGCTTTTATGGTTTCTGAGAACAGATGGGGCA-3′; and for 18S, Fwd 5′-AACCCTTGAACCCATT-3′, and Rev 5′-CCATCCAATCGGTAGTAGCG-3′.

### 2.12. Statistical Analysis

*P* was calculated by Student’s *t*-test from data presented as mean ± standard deviation (SD). Significant differences were accepted at *P* ≤ 0.05, as indicated.

## 3. Results

### 3.1. The LCR of HPV16 Contains Putative Binding Sites for SOX2

In order to identify transcription factor binding sites (TFBS) specific for HPV16- or HPV18-LCRs, we performed an in silico prediction analysis of probable TFBS using the JASPAR CORE database and alignment with consensus sequences. The results showed that although HPV16- and HPV18-LCRs shared TF binding elements, a group of sites that were restricted to each LCR, was identified. From this group, we found binding elements related to TFs involved in cellular processes such as proliferation and differentiation: FOX family (forkhead box); xenobiotic metabolism: Arnt (aryl hydrocarbon receptor nuclear translocator), FOXA2 (forkhead box); apoptosis: Ddit3 (DNA damage-inducible transcript 3); embryogenesis and tissue specificity: FOXD1, FOXF2, SOX2, MAFb (musculoaponeurotic fibrosarcoma homolog B) (Table 1).

**Figure 1:**
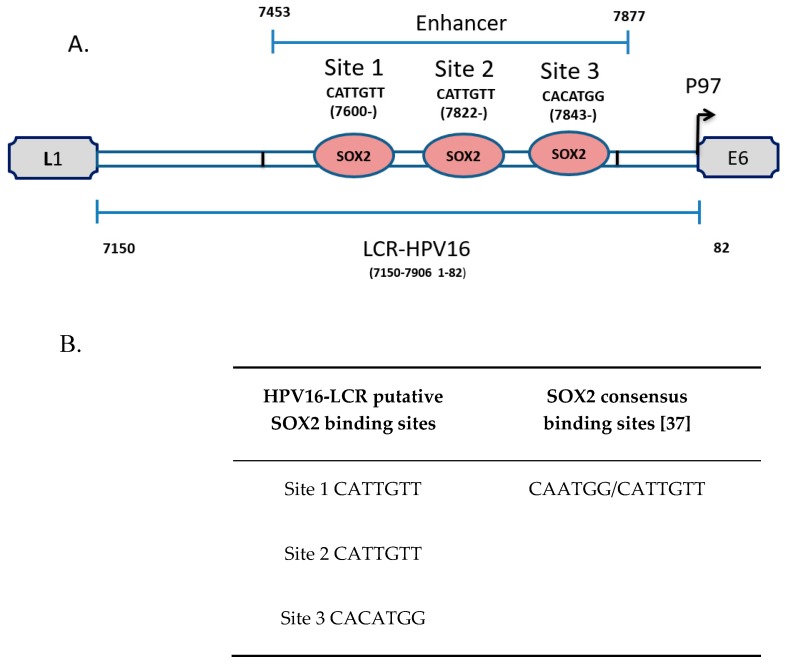
The LCR enhancer region of HPV16 contains sex determining region Y (SRY)-box 2 (SOX2) binding sites. (**A**) The nucleotide positions of the identified putative SOX2 binding sites (Site 1, Site 2 and Site 3) on the LCR enhancer region are shown; (**B**) Comparison of putative SOX2 binding sites to the reported SOX2 consensus sequences [37].

The presence of SOX2 TFBS, found exclusively in the enhancer region of HPV16-LCR, was particularly interesting since SOX2 participates in stem cell pluripotency [33], squamous differentiation [34], and in cervical carcinogenesis [35,36]. In our analysis, 3 putative binding SOX2 response elements were found in the HPV16-LCR: Site 1, Site 2 and Site 3 (Figure 1A). These sites were similar to the reported SOX2 consensus sequence (Figure 1B) [37]. We became interested in analysing the role of SOX2 in HPV regulation since there were no previous reports indicating its function in this context.

### 3.2. HPV16- and HPV18-LCRs Show Different Transcriptional Activities in Squamous Carcinoma and Adenocarcinoma Cell Lines

**Figure 2:**
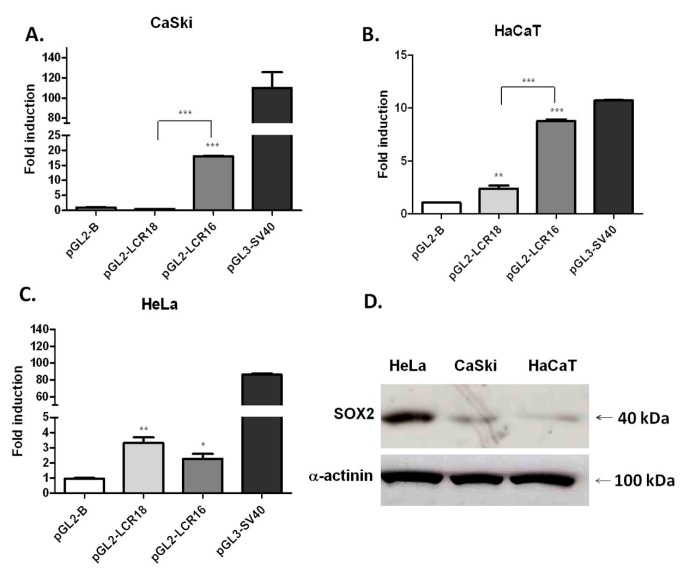
HPV16- and HPV18-LCRs present a different transcriptional activity in cells with distinct histological origin. Reporter plasmids were transfected in CaSki (**A**), HaCaT (**B**) and HeLa (**C**) cell lines. The transcriptional activity is shown as the fold induction, compared to the pGL2-B vector. The standard error of the mean is shown; * *p* < 0.05, ** *p* < 0.01, *** *p* < 0.001. The square brackets show differences between transcriptional activities of LCRs from HPV16 and HPV18. pGL3-simian virus 40 (SV40) plasmid harboring SV40 promoter was used as a positive control. The graphs show representative experiments carried out in triplicate and repeated three times; (**D**) Representative western blot of SOX2 basal protein levels in HeLa, CaSki and HaCaT cell lines. For loading control α-actinin was used.

The regulation of HPV transcriptional activity during the viral life cycle may be influenced by variations in the amounts of TFs or cofactors along the stratified epithelium, as well as under different cellular contexts. In order to determine the effect of the cellular context (squamous or glandular) on the transcriptional activity of HPV16- and HPV18-LCRs, promoter-reporter assays were performed in CaSki, HaCaT and HeLa cell lines with reporter vectors containing the luciferase gene under the control of LCR16 or LCR18. A significant activity of pGL2-LCR16 compared to the control pGL2-B vector was observed in the three cell lines (Figure 2A–C). Nevertheless, the activity was considerably higher in cells with a squamous cell origin (SQ), with a mean 19 and 8.3 times higher than the activity of the pGL2-B control vector in CaSki and HaCaT cells, respectively (Figure 2A,B). Additionally, LCR16 activity in HeLa cells, although significant, was only 2.4 times above the control (Figure 2C). 

In contrast, LCR18 activity was significantly lower than that of LCR16 in CaSki cells (Figure 2A). In HaCaT, LCR18 activity was nearly double that of the control, but 3.7 times lower than LCR16 (Figure 2B). Furthermore, in HeLa cells LCR18 activity was 3.5 times above the control, and although not significant, it was slightly higher than that of LCR16 (Figure 2C). As expected for a positive control, the SV40 promoter presented a high activity in CaSki and HeLa cell lines (116 and 90 times above the control, respectively) (Figure 2A,C); while in HaCaT cells it was 10 times above pGL2-B control vector (Figure 2B). This is probably due to lower transfection efficiencies commonly seen in the HaCaT cell line compared to the others [38]. However, it is clear that the activity of LCR16 in HaCaT cells is similar to that of the SV40 positive control. These results show a different behavior for HPV16- or HPV18-LCR depending on the cellular context of the tumor of origin (SQ or glandular). For LCR16, its transcriptional activity is favored in squamous epithelium, possibly from variations in the ratio of activating versus repressive factors. Conversely, the differences in transcriptional activity between HPV types could be due to variations in number, position or affinity of shared TFBS; or to TFBS which are present in one LCR, and absent in the other.

We analyzed endogenous SOX2 protein levels in the different cell lines by western blot. As shown in Figure 2D, HeLa cells expressed the highest level of SOX2, followed by CaSki; whereas HaCaT presented the lowest SOX2 protein levels (Figure 2D). Although many factors could be involved in the observed LCR transcriptional differences among our cell lines, it is noteworthy that the lower levels of SOX2 protein coincide with a relatively higher transcriptional activity of LCR16.

### 3.3. SOX2 Binds to the HPV16-LCR

#### 3.3.1. SOX2 Binds to Its Putative Binding Sites in LCR16 In Vitro

**Figure 3:**
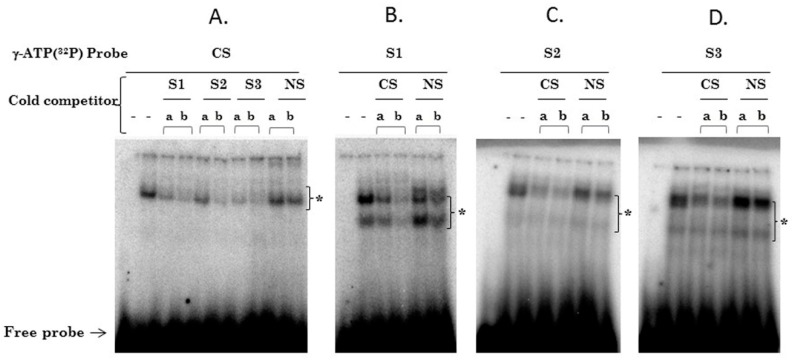

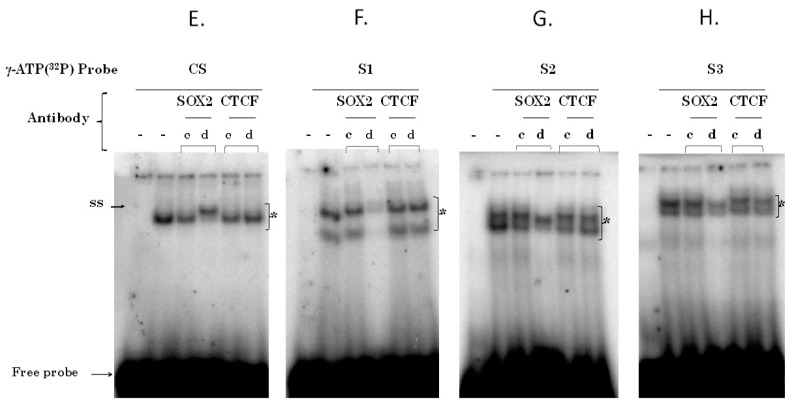
SOX2 binds to the putative SOX2 elements in HPV16-LCR. HeLa nuclear protein extracts were used in electrophoretic mobility shift assay (EMSA), to analyze the interactions between SOX2 and double-stranded DNA probes containing the three putative SOX2 binding sites (S1, S2 and S3) and the consensus binding site (CS). The first lanes of all the EMSAs are without nuclear extract; all the other lanes contain 15 μg of nuclear extract. Competitions with 60 (a) or 260 (b) fold excess of S1, S2, S3, NS (non-specific) and CS cold probes are shown (**A**–**D**). DNA-protein complexes are denoted by (*). Free-labeled probes are shown at the bottom of the EMSAs. EMSAs **E**–**H** show the DNA-protein complexes with the respective antibodies added (anti-SOX2 or anti-CCCTC-binding factor (CTCF), as indicated) at 50 ng (c) or 2500 ng (d). SS is the supershift formed with the CS probe and 2500 ng (d) of anti-SOX2 antibody (E). In **F**–**H**, a clear decrease in the DNA-protein complexes from each probe (S1, S2 and S3) was observed with 2500 ng (d) of anti-SOX2.

To determine whether SOX2 recognizes the three putative SOX2 response elements found in the HPV16-LCR, EMSA was performed using nuclear proteins from HeLa cells. The formation of one major DNA-protein complex (*) was observed when nuclear extracts were incubated with the SOX2 binding consensus probe (CS), labelled with γ-ATP (^32^P) (Figure 3A). In order to evaluate if the S1, S2 and S3 sites are able to compete with the DNA-Protein complex generated by the CS, different amounts of cold probes from S1, S2 or S3 were used. The complex formation with CS probe was strongly competed with the addition of 60 (a) and 260 (b) fold excess of the three cold probes; while cold non-specific oligonucleotide (NS) did not compete (Figure 3A). For S1, S2 or S3 probes we observed two major DNA-protein complexes (*) (Figure 3B–D). A similar behavior was observed when SOX2 sites (S1, S2 and S3) were inversely competed against a 50 (a) and 250 (b) fold excess of CS cold probe, where a clear drop in the DNA-protein complexes was observed, which was not observed with the NS cold probe (Figure 3B–D). These data suggest that SOX2 can bind to the three proposed binding sites found in the HPV16-LCR. 

In order to demonstrate that SOX2 protein is present in the DNA-protein complex, a supershift assay was performed. The presence of SOX2 in the DNA-protein complexes was confirmed after the addition of 50 ng (c) or 2500 ng (d) of anti-SOX2 antibody, resulting in a supershift band (SS) in the CS complex when using 2500 ng of antibody (d) (Figure 3E). A significant reduction of the DNA-protein complexes of S1, S2 and S3 probes was observed with 2500 ng (d) of SOX2 antibody (Figure 3F–H). The specificity of SOX2 antibody was confirmed, since no change occurred with 50 or 2500 ng (c and d) of anti-CTCF antibody (Figure 3E–H). Thus, our in vitro results support the novel interaction of SOX2 with the three putative bindings sites present in the HPV16-LCR.

#### 3.3.2. SOX2 Binds to the HPV16-LCR In Vivo

In order to confirm that SOX2 binds to the HPV16-LCR in vivo, ChIP assays were performed with chromatin extracts obtained from CaSki cells. ChIP results demonstrate that SOX2 binds in vivo to the HPV16-LCR. As a positive control we employed anti-p65 antibody, since it is known that p65 binds to HPV16-LCR [31]. Previously, it has been reported that CTCF is not recruited to HPV16-LCR, therefore we used anti-CTCF antibody as a non-binding control [32]. 

**Figure 4:**
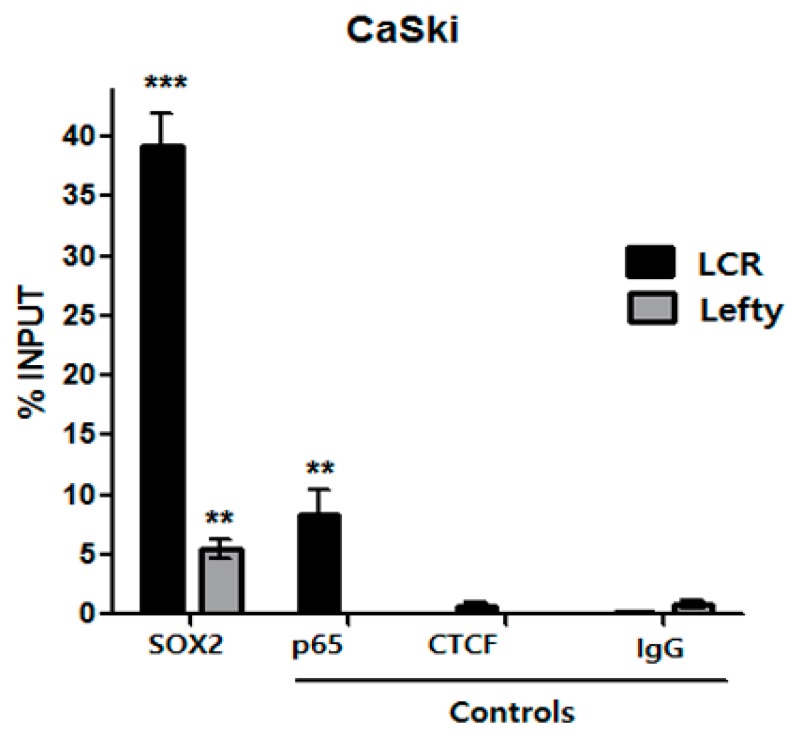
SOX2 binds to HPV16-LCR putative binding sites in vivo. Chromatin immunoprecipitation assay (ChIP) of SOX2 were performed evaluating the HPV16-LCR region (LCR) from CaSki cells. Anti-p65 and -CTCF antibodies were employed as positive and negative controls, respectively. Non-specific IgG antibody was included as a negative control. Quantitative PCR (qPCR) analysis was performed on the DNA obtained from the ChIP assay evaluating the HPV16-LCR region (black bars), and the Lefty (gray bars) gene promoter region, as a positive control of SOX2 binding (*n* = 2). The graphic represents the percentage of input recovered after immunoprecipitation with anti-SOX2, -p65 or -CTCF antibodies. Three independent biological experiments were performed. The standard error of the mean of triplicate qPCR measurements is shown. ** *p* < 0.01, *** *p* < 0.001.

ChIP results show that the DNA fragment containing the HPV16-LCR SOX2 binding sites was qPCR-amplified from the anti-SOX2 immunoprecipitated sample, with near 40% above IgG background (Figure 4). Such enrichment was higher than the 8% observed when immunoprecipitating with anti-p65 antibody. When using anti-CTCF as a negative control, there was no important LCR amplification. The proportion of SOX2 binding to the LCR is even higher than that to its target Lefty (5%), used as positive control of SOX2 binding. These results demonstrate that SOX2 binds to the HPV16-LCR in vivo.

### 3.4. SOX2 Represses HPV16-LCR Transcriptional Activity

**Figure 5:**
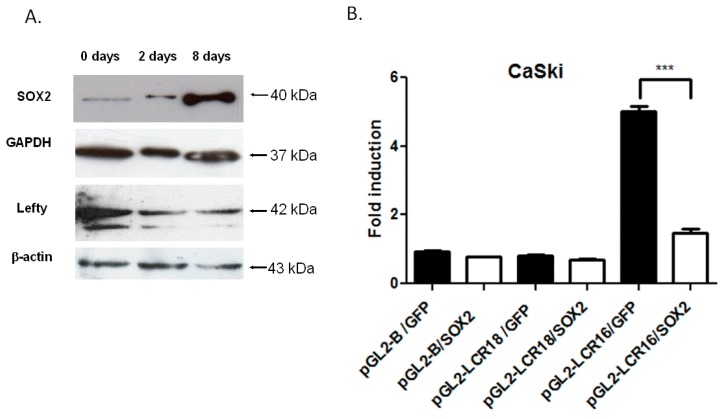
SOX2 represses the transcriptional activity of HPV16-LCR. (**A**) SOX2 was overexpressed in CaSki cells using a lentiviral system. Shown is a representative western blot of the expression levels of SOX2 and its target Lefty, at different days post-transduction (0, 2 and 8 days). Glyceraldehyde-3-phosphate dehydrogenase (GAPDH) and β-actin antibodies were used as protein loading controls; (**B**) CaSki cells transduced with lentiviral vectors expressing SOX2 and/or green fluorescent protein (GFP) were transfected with luciferase reporter plasmids containing LCR16 or LCR18. The transcriptional activity is shown as fold induction, compared to the pGL2-B/GFP vector. The graph shows a representative experiment carried out in triplicate and repeated three times. The standard error of the mean is shown. *** *p* < 0.001.

In order to elucidate the effect of SOX2 on the transcriptional regulation of HPV16-LCR, CaSki cells were transduced with lentiviral vectors carrying SOX2, and/or GFP as control. After 24 h, cells were transfected with reporter plasmids pGL2-LCR16 or pGL2-LCR18. Cells were processed 48 h later. To assess the presence of a functional overexpressed SOX2, protein levels of Lefty, a known SOX2 gene target, were also measured at different days post transduction. As expected, Lefty protein levels decreased as SOX2 increased (Figure 5A). At 48 h post-transfection, significant repression of the LCR16 was observed due to SOX2 overexpression, remaining 3.5 times beneath the GFP transduced cells (Figure 5B). LCR18 activity was not affected by SOX2 overexpression. These results indicate that SOX2 is acting as a repressor of LCR16 in CaSki cells.

### 3.5. HPV16-LCR Sites S1, S2 and S3 Are Required for SOX2 Mediated Repression

**Figure 6:**
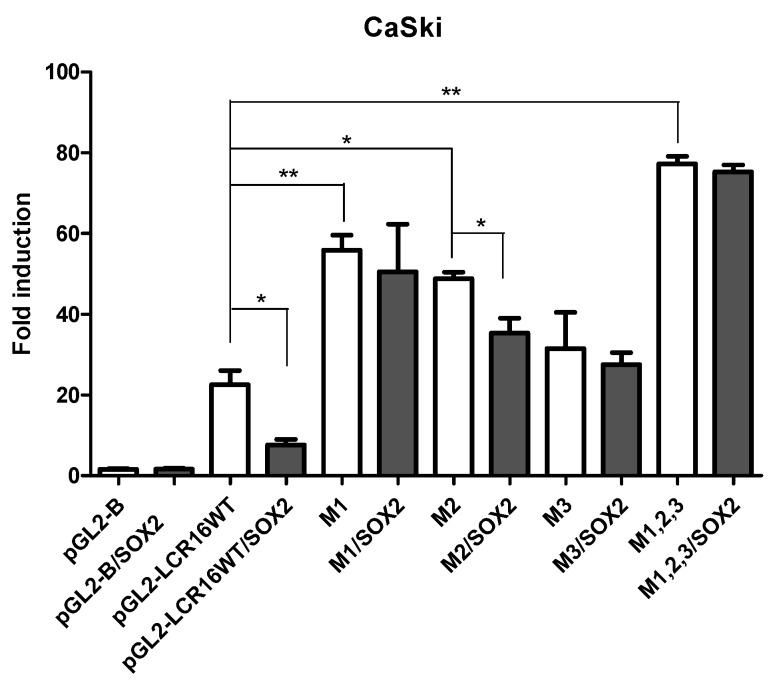
HPV16-LCR sites S1, S2 and S3 have a role in SOX2 repressor activity. Site directed mutagenesis was performed on the HPV16-LCR (wt, wild type) plasmid, generating M1 (LCR-Site 1 mutant), M2 (LCR-Site 2 mutant), M3 (LCR-Site 3 mutant) and M1,2,3 (LCR triple mutant) mutants. Transcriptional activity of luciferase reporter plasmids was measured in CaSki cells in the presence (black bars) or absence (white bars) of ectopically expressed SOX2. Fold induction of LCR16 activity was calculated in relation to the control vector pGL2-B. LCR16 mutant transcriptional activities were compared with pGL2-LCR16wt activity (white bars). Transcriptional activity of each LCR16 (wt, M1, M2, M3, or M1,2,3) was compared to its respective condition with and without SOX2 (white vs. black bars). Standard error of the mean is shown. * *p* < 0.05, ** *p* < 0.01.

To test whether SOX2 binding to S1, S2 and S3 sites is involved in the observed LCR16 transcriptional repression, mutations were generated in the pGL2-LCR16 vector (wt, wild type): 2-base pairs (bp) mutated in S1 site (M1), 3-bp mutated in each of the S2 and S3 sites (M2 and M3, respectively), and 8-bp mutations are introduced in the triple mutant (M1,2,3) (Figure S1). As shown through luciferase activity, a recovery of LCR16 transcriptional activity, compared to LCR16wt, is clearly observed with M1, M2 and M1,2,3 (white bars), suggesting that the respective mutations escape from the repressive effects of endogenous SOX2. To determine if SOX2 is involved in this effect, the protein was ectopically overexpressed (black bars); LCR16wt activity was repressed up to 66.2% and M1, M3 and M1,2,3 were not repressed by SOX2 overexpression; while M2 was mildly repressed in a proportion of 27.5%. These data confirmed that the three SOX2 putative binding sites S1, S2, and S3, contribute to SOX2 repressor activity (Figure 6) and that there is a direct interaction of SOX2 with the three sites.

### 3.6. E6 and E7 Endogenous Expression Is Down Regulated by SOX2

**Figure 7:**
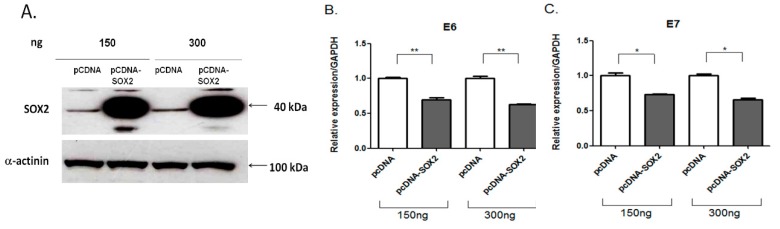
SOX2 represses endogenous *E6* and *E7* viral oncogenes expression. CaSki cells were transfected with 150 or 300 ng of SOX2 expressing plasmid. (**A**) SOX2 protein levels after transfection are shown. For protein loading control, α-actinin levels were analyzed. Quantitative Reverse transcription-polymerase chain reaction (qRT-PCR) of endogenous E6 (**B**) and E7 (**C**) viral oncogenes in the presence or absence of overexpressed SOX2, was performed. The empty vector pcDNA was used as a control. *E6* and *E7* expression levels were significantly diminished in the presence of SOX2. Data were normalized to the housekeeping gene GAPDH. Standard error of the mean is shown. * *p* < 0.05; ** *p* < 0.01.

Since *E6* and *E7* viral oncogenes are directly regulated by the LCR, we next analyzed whether SOX2 was acting as a transcriptional repressor of such oncogenes in vivo. Two amounts of SOX2 transcription factor expressing plasmid (150 and 300 ng) were transfected into CaSki cells to analyze its effect on the endogenous levels of *E6* and *E7* transcripts (Figure 7). As shown in the western blot, SOX2 protein was overexpressed in SOX2 transfected cells (7A). Quantitative PCR analysis shows that E6 (7B) and E7 (7C) transcript levels were significantly decreased in the presence of SOX2 (about 40 to 33%, respectively). No SOX2 dose-dependent repression of E6 and E7 was observed.

## 4. Discussion

Several factors, including host, cellular or viral features, could determine the high prevalence of HPV16 observed in squamous cell carcinoma of the cervix, in relation to other oncogenic HPVs. Among them, it is possible that a set of specific transcription factors could differentially guide viral transcriptional activity in the different cell lines. To investigate the effect of the cellular context in the transcriptional activity of HPV16- and 18-LCRs, we performed assays with reporter plasmids. We found that LCR16 activity was significantly higher than that of LCR18 in CaSki and HaCaT cell lines, which have a SQ context, possibly due to a particular set of TFs shared in squamous cells. In contrast, in the adenocarcinoma HeLa cell line, LCR transcriptional activities are lower but similar for both HPV16- and 18-LCRs. This suggests that SQ context may favor LCR16 activity or that a glandular context (TFs found in cells with glandular origin) may repress it. A differential activity between LCR16 and LCR18 in the three cell lines was evident, which could be due in part to differences in number, position or affinity of shared TFBS, or to the presence of different TFBS for each LCR.

To identify novel TFs that could modulate the transcriptional activity of LCR16 and LCR18, we searched for putative binding sites for specific TFs. As expected, we found some TFBS shared between both viral types; however, there were others specific for each of the LCRs. We became interested in SOX2 transcriptional factor; due to the fact that consensus-binding sites for this factor were exclusively identified in the HPV16-LCR sequence and that it is involved in normal development as well as in malignant processes [39]. This TF is a member of the SOX (sex determining region Y (SRY)-box) transcriptional factor family and is a key controller of embryogenesis and cell fate during development [40], additionally participating in the maintenance of pluripotency [33]. Although the predominant role of SOX2 has been described as a transcriptional activator, it has recently been shown to play a dual role, also acting as a repressor of gene expression [41,42].

SOX2 has been linked to cancer hallmarks, promoting cell proliferation in breast, pancreatic, prostate and cervical cancers [36,43,44,45,46], as well as evading apoptosis in prostate, gastric and lung cancers [45,47,48]. Furthermore, SOX2 expression has been associated with early events of cervical carcinogenesis [49], since it is highly expressed in premalignant lesions, as well as in cervical cancer, while its expression is low in normal cervical epithelium. In addition, SOX2 overexpression in cervical cancer cell lines causes an increase in proliferation, clonogenicity and tumorigenicity [36]. These results suggest that SOX2 has an essential role in cervical carcinogenesis. Nevertheless, the effect of SOX2 in the transcriptional regulation of high-risk HPVs has not been previously studied. 

In this study, we demonstrated that SOX2 binds to three putative binding sites identified in the enhancer sequence of LCR16 through direct interactions, as shown in EMSA and ChIP assays. We also confirmed that SOX2 is a transcriptional repressor of LCR16, since its activity decreased 3.5 times when SOX2 was over-expressed in CaSki cells (Figure 5). As has been previously reported, HPV16- and HPV18-LCRs share a repertoire of TFBS regulating their activity [28]; however, TFBS that are specific for each LCR type may partly explain the epidemiological data related to the different distribution of oncogenic HPV types, depending on cellular histological characteristics.

The direct interaction of SOX2 on the LCR16 putative binding sites was demonstrated through the analysis transcriptional activities of the mutated binding sites. A significant recovery of luciferase activity was observed with M1, M2 and M1,2,3 mutants in the basal state, which suggested that LCR16 mutants escaped from endogenous SOX2 repression. Moreover, when SOX2 was ectopically overexpressed, no repression of LCR16 M1 and M3 was observed and M2 mutant showed a mild repression (27.5%), in contrast to the observed for LCR16wt (66.2%). These data demonstrate that the three SOX2 putative binding sites contribute to the repressor activity of SOX2 in the HPV16-LCR.

SOX2 repressor activity on LCR16 was confirmed in vivo in CaSki cells which contain HPV16 endogenous sequences, where E6 and E7 oncogenes are naturally expressed. Quantitative PCR analysis showed that E6 and E7 transcript levels significantly decreased in cells overexpressing SOX2, although no dose dependent effect of SOX2 was observed, probably due to a saturation of the putative sites at the tested SOX2 doses. With these data, we demonstrate a direct effect of SOX2 on HPV16-LCR, although we cannot discard the contribution of indirect factors. Bass et al. (2009), showed that an increase in the expression of various stem cell TFs was correlated with high levels of SOX2 expression in SCC lung tumors [34]; therefore, we cannot rule out the possibility that other transcription factors, regulated by SOX2, could also contribute to the modulation of LCR activity.

It is interesting that the analysis of SOX2 protein levels in the different cell lines showed that HeLa cells had the higher SOX2 protein levels, compared to the SCC lines (CaSki and HaCaT). These data match the higher transcriptional activities observed for LCR16 in the SQ context, in which HPV16 transcription could be favored due to a less repressive context. Therefore, under this background, it is possible that SOX2 is not acting as an activator of the LCR16 in SCC cells, but instead, as a repressor in adeno cells. Hence, high levels of SOX2 in HeLa cells could have a participation in the low activity observed for LCR16 in this cell type. However, it is important to consider that TFs that we did not analyze in this work surely influence the activity of the HPV LCRs.

It is important to note that even though in our study we did not delve further into the distinct transcriptional activities of HPV18-LCR observed in the analyzed cell contexts, it is a subject that deserves additional studies. The assays with reporter vectors showed a clear down-regulation of the LCR18 under a SQ cell context. Furthermore, the in silico analysis also showed putative TFs binding sites that were specific for HPV18-LCR. Thus, it would be relevant to study which novel transcriptional factors could be modulating LCR18 activity.

SOX2 regulation of HPV16-LCR could be biologically relevant in early HPV infections. HPV may infect basal as well as stem cells [50,51], where an overexpression of SOX2 in the latter would be expected. In fact, high levels of SOX2 have been demonstrated in cervical cancer [52] and particularly, in a subpopulation of tumor initiating cells with stem-like properties [53]. In such scenario, a controlled expression of E6 and E7 oncogenes, not only through viral but also through cellular proteins such as SOX2, may retard cellular damage and hence, viral persistence may be favored. The study of this landscape could help to explain, in part, the high prevalence of HPV16 in squamous cell carcinoma.

It is worth mentioning that in cervical SCCs a high expression of SOX2 has been reported; however, the loss of SOX2 in a subgroup of cervical cancer cases, has been associated with poor prognosis [49]. Furthermore, it has been shown that the increase in HPV oncogene mRNA expression is associated with poor prognosis in cervical cancer patients [54]. Consequently, if SOX2 acts as a transcriptional repressor of HPV16-LCR, its down-regulation in cancer cells could eventually promote an increase in the LCR activity, reflected as an overexpression of the E6 and E7 oncogenes. To further study this possibility, it would be necessary to determine whether in HPV16 positive cervical cancer biopsies the amount of SOX2 is inversely associated with the expression levels of E6 and E7 oncogenes. In this situation, it should also be considered that SOX2 associates with many partner proteins, including co-activators and co-repressors, and such diverse associations may differentially modulate target genes [55]. In neural stem cells it has been shown that when SOX2 acts as a repressor, it requires the interaction with members of the Groucho family of repressors [41]. Therefore, the analysis of such possible interactions in cervical cancer cells could provide relevant information for understanding the mechanisms of transcriptional regulation of HPV by SOX2.

In this study, we identified SOX2 as a new player in the transcriptional control of HPV16. A greater comprehension of the differential regulation of HPV types according to the cellular context will help to explain differences in their biological behavior.

## Figures and Tables

**Table 1:** In silico prediction of transcription factor binding sites exclusive for HPV16- or HPV18-LCR.

	LCR-HPV16			LCR-HPV18	
Arnt	TGTTGTTTGTTG	(nt 7180)	Ddit3::Cebpa	TGATTG	(nt 7205)
	TGTTGTTTGTT	(nt 7180)	Gata1	GTACTGTA	(nt 7504)
Foxa2	CACGTG	(nt 7268)	MZF1-1-4	CAATTGCT	(nt 7639)
	CGTGTGTA	(nt 7270)	NFE2L2	ATAGTTA	(nt 7651)
	GTGTATGTGTTT	(nt 7273)	NKX3-2	TAATTGCA	(nt 7720)
FoxD1	TGACACAAT	(nt 7303)		AATTA	(nt 7720)
	TGACACA	(nt 7305)	TAL1::TCF3	AGTATATA	(nt 22)
FoxF2	ATAAGTT	(nt 7325)			
HNF1B	TTTCCT	(nt 7569)			
	TCAGGAA	(nt 7570)			
MAFb	TTCCATTGTT	(nt 7597)			
MAX	TCCATTGTT	(nt 7598)			
Mycn	GTAAAAAA	(nt 7605)			
SOX2	CATTGTT	(nt 7600)			
	CATTGTT	(nt 7822)			
	CACATGG	(nt 7843)			

Transcription factor binding sites (TFBS) exclusive for each long control region (LCR). The sequence and nucleotide (nt) positions of putative TFBS are identified in human papillomavirus (HPV) 16 and HPV18 LCR sequences. Arnt (aryl hydrocarbon receptor nuclear translocator); Fox A2, Fox D1, Fox F2 (forkhead box); HNF1B (hepatocyte nuclear factor 1 homeobox B); MAFb (musculoaponeurotic fibrosarcoma homolog B); MAX (Myc-associated factor X); Mycn (avian myelocytomatosis viral oncogene neuroblastoma); SOX2 (sex determining region Y (SRY)-box 2); DDIT3 (DNA damage-inducible transcript 3); Cebpa (CCAAT/enhancer-binding protein α); GATA1 (globin transcription factor 1); MZF1-1-4 (myeloid zinc finger 1); NFE2L2 (nuclear factor erythroid 2 like 2); NKX3-2 (homeobox protein NK-3 homolog B); TAL1 (T-cell acute lymphocytic leukemia protein 1); TCF3 (transcription factor 3).

## Supplementary Materials

The following are available online at www.mdpi.com/1999-4915/9/7/175/s1, Figure S1: Identification of the nucleotides mutated in the Long Control Region (LCR) from HPV-16 by multi site-directed mutagenesis. Table S1: Primers used to introduce mutations in the putative SOX2 binding sites 1, 2 and 3 of the HPV16-LCR.
